# Monocyte USP7-p65 axis mediates immune responses to the immunogenicity of nucleus pulposus

**DOI:** 10.1016/j.cstres.2025.100114

**Published:** 2025-09-12

**Authors:** Peng Feng, Xuelei Chu, Ying Che, Jinghua Gao, Chunyu Gao, Ting Zhang

**Affiliations:** 1 Department of Orthopaedic Surgery, Wangjing Hospital of China Academy of Chinese Medical Sciences, Beijing 100700, China; 2 Department of Orthopaedic Surgery, University of Pittsburgh, Pittsburgh, PA 15213; 3 Department of Oncology, Guang'anmen Hospital, China Academy of Chinese Medical Sciences, Beijing 100053, China; 4 School of Medicine, Shandong University of Traditional Chinese Medicine, Jinan 250355, China

**Keywords:** Nucleus pulposus, Immunogenicity, Monocytes, Intervertebral disc degeneration, USP7, P65

## Abstract

The nucleus pulposus (NP) of the intervertebral disc is an immune-privileged tissue. During intervertebral disc degeneration (IDD), this immune privilege is compromised, resulting in the exposure of NP components to the peripheral immune system, which in turn activates monocytes and elicits an immune response. In this study, we demonstrate that monocytes respond to NP immunogenicity by activating damage-associated molecular patterns (DAMPs), thereby initiating a sustained NF-κB–mediated inflammatory response in NP tissue and ultimately driving a vicious cycle of inflammation and oxidative stress within NP cells. Mechanistically, NP-derived immunogenic stimulation induces monocyte activation, accompanied by increased expression and nuclear translocation of the deubiquitinase USP7. USP7 promotes the accumulation and nuclear translocation of the NF-κB subunit p65 via a deubiquitination-dependent mechanism, leading to enhanced transcription of TNF-α, HMGB1, and IL-1β. These DAMP-associated cytokines further stimulate NP cells, resulting in upregulation of HMGB1, TNF-α, COX-2, IL-1β, and reactive oxygen species (ROS), along with a concomitant decrease in the antioxidant enzyme SOD2—collectively amplifying inflammation and oxidative stress within the NP microenvironment. Dual-luciferase reporter assays and chromatin immunoprecipitation (ChIP)-qPCR demonstrated that knockdown of USP7 in monocytes significantly reduced p65 binding to the promoter regions of TNF-α, HMGB1, and IL-1β, thereby attenuating the downstream inflammatory and oxidative stress responses in NP cells. Together, these findings uncover a novel immune-inflammatory mechanism underlying IDD and highlight the USP7-mediated pathway in monocytes as a potential therapeutic target for modulating disc degeneration.

## Graphical abstract

**Figure:**
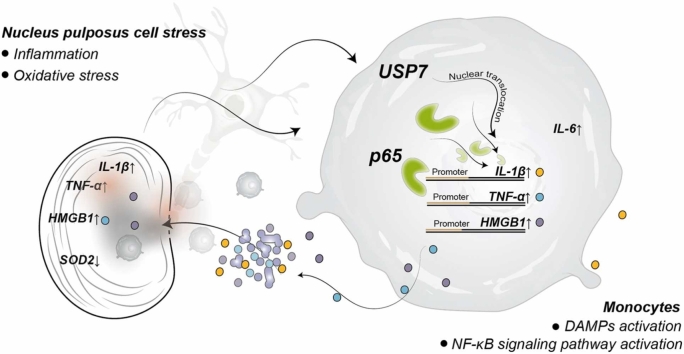

## Introduction

Low back pain (LBP) has emerged as the leading cause of disability worldwide, imposing a tremendous public health burden.^1^ Intervertebral disc degeneration (IDD) is one of the most common causes of chronic LBP, contributing to an estimated 40% of chronic LBP cases in adults.^2^ As the global population ages, the prevalence of degenerative spine conditions continues to rise, with hundreds of millions of people affected and significant socioeconomic losses from reduced work capacity.^1^ These epidemiological trends underscore the urgent need to better understand IDD pathogenesis and develop effective therapies.

The nucleus pulposus (NP), the gelatinous core of the intervertebral disc, originates from the embryonic notochord and is normally an immune-privileged tissue isolated from the body’s immune system by the surrounding annulus fibrosus and cartilage endplates.^3^ In a healthy disc, the NP is avascular and sequestered from immune surveillance. However, when IDD progresses and the annulus fibrosus is compromised (through fissures, herniation, or endplate damage), this “immune barrier” is broken and NP components become exposed to the immune system.^3^, ^4^, ^5^, ^6^ The formerly sequestered NP is then recognized as a foreign antigen, triggering an immune response in the disc.^7^, ^8^ Infiltrating immune cells—predominantly monocytes and macrophages—are recruited to the NP and release a variety of pro-inflammatory cytokines (e.g., IL-1β, IL-6, TNF-α, IFN-γ) and chemokines that create an inflammatory cascade, as well as matrix-degrading enzymes like matrix metalloproteinases (MMPs) that break down the extracellular matrix.^3^ This immune-mediated attack exacerbates the structural damage and functional deterioration of the disc. Indeed, NP immunogenicity is now recognized as a key factor precipitating these immune reactions in IDD—inert disc components or intact annulus tissue will not elicit macrophage recruitment, whereas exposed NP readily attracts immune cell infiltration.^9^, ^10^ Recent in vivo experiments further underscore the pathological impact of NP immunogenicity: for example, immunizing animals against NP antigens to pre-sensitize the immune system led to a markedly accelerated disc degeneration following injury, compared to controls without NP immunization.^7^ These findings highlight that the NP’s loss of immune privilege and the ensuing immune response are central events in IDD progression.

Among the various immune cells involved, monocytes and their derivative macrophages appear to play a pivotal role in the NP-induced inflammatory cascade.^11^ Upon NP exposure, chemotactic signals such as monocyte chemoattractant protein-1 (MCP-1/CCL2) attract circulating monocytes into the disc, where they differentiate into pro-inflammatory M1 macrophages.^12^ They also release enzyme-rich phagolysosomal vesicles containing MMPs and other proteases that actively degrade NP tissue.^13^ However, the molecular mechanism by which NP immunogenicity triggers monocyte activation and pro-inflammatory polarization remains incompletely understood. Therefore, in this study, we employed both an in vivo rat tail disc degeneration model and an in vitro NP–monocyte co-culture system to investigate how NP immunogenicity activates monocytes at the molecular level.

## Results

### Significant infiltration and activation of monocytes in degenerated rat intervertebral discs

**Fig. 1:**
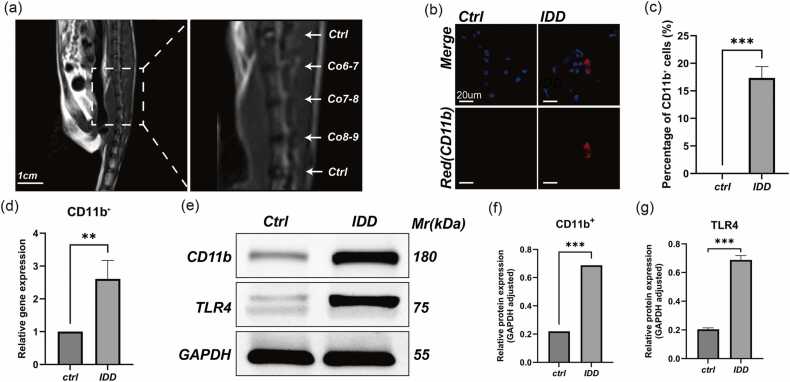
*Significant monocyte infiltration and activation in degenerated rat intervertebral discs.* (a) Representative MRI image obtained 4 weeks after needle puncture of rat tail intervertebral discs. The right panel displays a magnified view of the highlighted region in the left panel. Scale bar: 1 cm. (b) and (c) Immunofluorescence staining of CD11b⁺ monocytes in the nucleus pulposus (NP) region (b) and quantification of CD11b⁺ cells (c). CD11b⁺ signals are shown in red and nuclei are counterstained with DAPI (blue). Quantification is expressed as the percentage of CD11b⁺ cells relative to total DAPI⁺ nuclei. Scale bar: 20 µm *n* = 3. (d) Quantitative PCR analysis of CD11b mRNA expression in NP tissues from normal and degenerated discs *n* = 3. (e-g) Western blot analysis of CD11b and TLR4 protein levels in NP tissues (e), with densitometric quantification shown in panel f and g *n* = 3. Data are presented as mean ± standard deviation (SD). Statistical significance was determined using an unpaired two-tailed Student’s *t* test. *P < 0.05, **P < 0.01, ***P < 0.001.

Following the establishment of a needle puncture–induced rat tail IDD model, we evaluated monocyte infiltration in the NP region via immunofluorescence staining. MRI imaging of punctured discs revealed typical features of “black disc disease,” showing clear degeneration compared to control discs (Figure 1a). Immunofluorescence analysis demonstrated that CD11b^+^ monocytes were scarcely detectable in the NP region of healthy discs, whereas a marked accumulation of CD11b⁺ cells was observed in the NP area of puncture-induced degenerated discs (Figure 1b and c). Consistently, qPCR analysis revealed significantly elevated mRNA expression of CD11b in degenerated NP tissues relative to controls, supporting the histological findings (Figure 1d). Western blot analysis further confirmed that the protein levels of CD11b and TLR4 were markedly upregulated in degenerated NP tissues (Figure 1e-g). These findings indicate that the immune-privileged status of the NP is disrupted during disc degeneration, facilitating the infiltration of peripheral monocytes and upregulation of their surface markers and pattern recognition receptors, suggesting robust activation of local damage-associated molecular pattern (DAMP)-associated signaling mechanisms.

### Single-cell RNA sequencing reveals markedly elevated USP7 expression in monocytes during disc degeneration

**Fig. 2:**
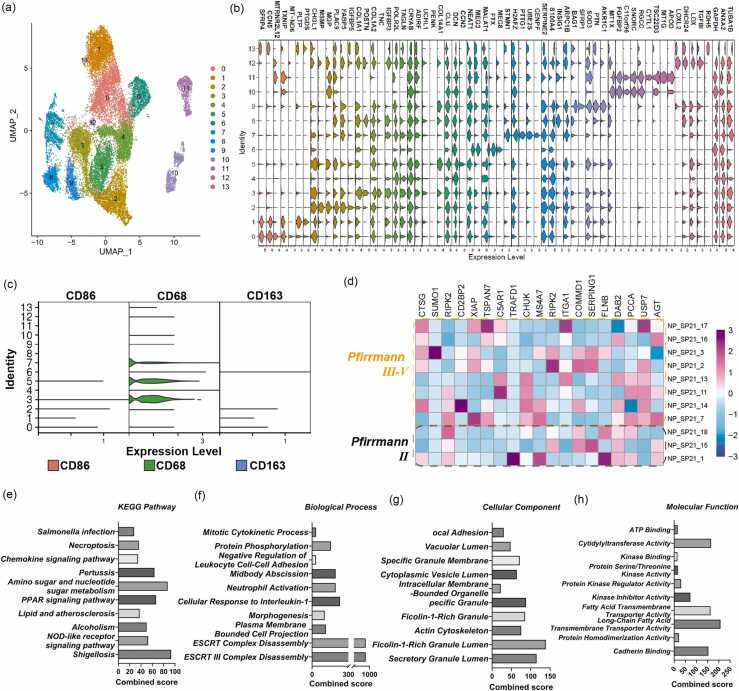
*Single-cell RNA sequencing reveals significantly elevated USP7 expression in monocytes.* (a) UMAP visualization of single-cell transcriptomic profiles. Each dot represents an individual cell, color-coded according to unsupervised clustering. A total of 14 distinct cell subpopulations (Clusters 0-13) were identified, reflecting the cellular heterogeneity within degenerated disc tissue. (b) Violin plots displaying the expression patterns of the top five marker genes for each cluster, facilitating cell type annotation based on transcriptional signatures. (c) Violin plots showing the expression of canonical monocyte/macrophage marker genes across all clusters. (d) Heatmap depicting the top 150 highly expressed genes in monocyte/macrophage populations derived from mildly, moderately, and severely degenerated tissues, highlighting changes in gene expression associated with disease severity. (e)-(h) KEGG and GO enrichment analyses of highly expressed genes in monocyte/macrophage clusters. KEGG pathways enriched in immune and inflammatory responses, including NOD-like receptor signaling, PPAR signaling, chemokine signaling, and necroptosis (e). GO enrichment results categorized into biological processes, cellular components, and molecular functions (f-h).

To elucidate the molecular characteristics of monocytes within the inflammatory microenvironment of IDD, we analyzed single-cell RNA sequencing data derived from human degenerated disc tissue (Table 1). Through unsupervised clustering of transcriptomic profiles from tens of thousands of individual cells, we identified distinct cellular subpopulations, including those enriched for monocyte-associated transcripts. These clusters exhibited high expression of CD68, a canonical marker indicative of a monocyte/macrophage phenotype (Figure 2a). We further compiled the top five most differentially expressed marker genes for each cluster to aid in their characterization (Figure 2b). Based on the expression of established monocyte/macrophage markers, clusters 3, 5, and 7 were classified as monocyte/macrophage populations (Figure 2c).

**Table 1:** Single-cell RNA sequencing data.^23^

	GEO Series Accession Number	GEO Sample Accession Number	Sample Name
Degenerated nucleus pulposus	GSE230808	GSM7235341	NP_SP21_007
	GSE230808	GSM7235342	NP_SP21_011
	GSE230808	GSM7235343	NP_SP21_013
	GSE230808	GSM7235344	NP_SP21_014
	GSE230808	GSM7235345	NP_SP21_016
	GSE230808	GSM7235346	NP_SP21_017
	GSE230808	GSM7235347	NP_SP22_002
	GSE230808	GSM7235348	NP_SP22_003
Normal nucleus pulposus	GSE229711	GSM7173751	NP_SP21_015
	GSE229711	GSM7173752	NP_SP21_018
	GSE229711	GSM7173753	NP_SP22_001

In this monocyte cluster, we found that USP7 expression was markedly elevated in monocytes derived from moderately and severely degenerated NP tissues, compared to those from mildly degenerated NP tissue (Figure 2d). KEGG pathway enrichment analysis of DEGs revealed significant enrichment in multiple immune- and inflammation-related pathways. Notably, pathways such as the NOD-like receptor signaling pathway, PPAR signaling pathway, and chemokine signaling pathway were prominently enriched, suggesting that disc degeneration is associated with innate immune recognition, lipid-mediated transcriptional regulation, and inflammatory cell recruitment—hallmarks of an enhanced local innate immune response in degenerated NP tissue (Figure 2e). Additionally, enrichment of the necroptosis pathway implies that programmed necrotic cell death may further amplify inflammation and contribute to tissue injury (Figure 2e). Gene Ontology functional analysis further supported these findings. In the biological process category, enriched terms included ESCRT complex disassembly, cell division, and neutrophil activation, indicating coordinated roles in membrane remodeling and immune response (Figure 2f). The cellular component category highlighted terms such as secretory granule lumen, granule membrane, and actin cytoskeleton, reflecting active vesicle trafficking and cytoskeletal reorganization (Figure 2g). In the molecular function category, significant enrichment was observed for cadherin binding, fatty acid transport, and kinase regulator activity, implicating processes related to cell adhesion, metabolic modulation, and intracellular signaling (Figure 2h).

Collectively, these results suggest that the DEGs in monocytes from degenerated NP tissues are involved in orchestrating immune-inflammatory responses and structural remodeling, thereby contributing to the pathophysiological progression of IDD.

### NP immunogenicity activates the monocyte USP7–p65 axis to induce NP cells inflammation and oxidative stress

**Fig. 3:**
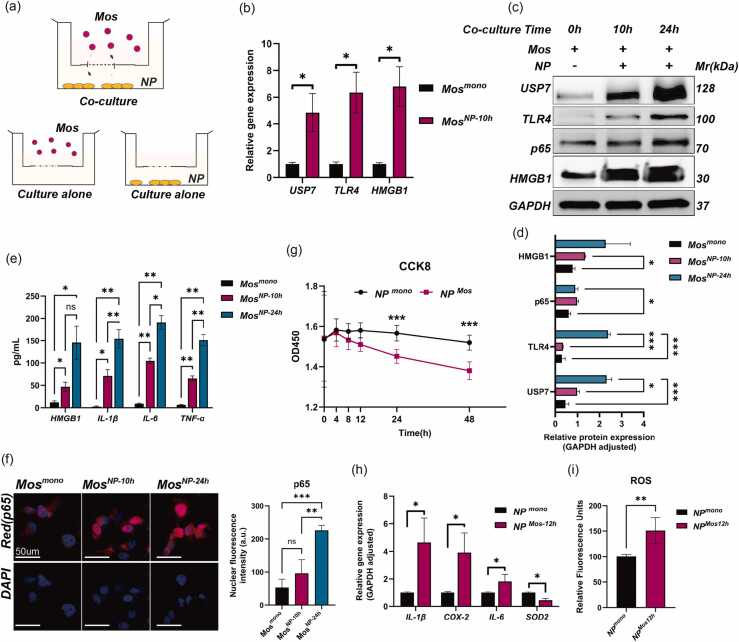
*NP cells activate monocytes, leading to stress responses in NP cells.* (a) Schematic illustration of the Transwell co-culture system used to assess interactions between NP cells and monocytes (Mos). Three culture conditions were established in the Transwell system: NP–Mos co-culture (referred to as *Mos*^*NP*^ or *NP*^*Mos*^, depending on the cell type being analyzed), Mos monoculture (*Mos*^*mono*^), and NP monoculture (*NP*^*mono*^). (b) qPCR analysis of USP7, TLR4, and HMGB1 mRNA expression in monocytes stimulated with or without NP cells. Data were analyzed using an independent samples *t* test. (*n* = 3). (c) and (d) Western blot analysis of USP7, p65, TLR4, and HMGB1 protein levels in monocytes cultured with or without NP cells for 10 or 24 h (c), with densitometric quantification shown in panel D. Data are presented as mean ± SD and were analyzed using two-way ANOVA (*n* = 3 or 4). (e) ELISA quantification of HMGB1, IL-1β, IL-6, and TNF-α levels in the culture medium under monoculture and co-culture conditions at different time points. Data were analyzed using two-way ANOVA (*n* = 3). (f) Immunofluorescence analysis of p65 nuclear localization and expression in monocytes under monoculture or co-culture conditions. Quantification is shown on the right. Data were analyzed using two-way ANOVA (*n* = 3). (g) CCK-8 assay assessing NP cell viability under monoculture and co-culture conditions at 0, 4, 8, 12, 24, and 48 hours. Statistical analysis was performed using two-way ANOVA (*n* = 5). (h) qPCR analysis of IL-1β, IL-6, COX-2, and SOD2 mRNA expression in NP cells cultured alone or co-cultured with monocytes. Data were analyzed using an independent samples *t* test (*n* = 3). (i) DCFH-DA fluorescence assay for ROS accumulation in NP cells under monoculture or co-culture conditions. Statistical significance was assessed using an independent samples *t* test. Data are presented as mean ± SD. *P < 0.05, **P < 0.01, ***P < 0.001. *n* = 3.

To further investigate the interaction between monocytes and NP cells, a Transwell co-culture system was established (Figure 3a). After 10 h of exposure to NP cell–conditioned medium, a time point selected to capture early transcriptional responses, monocytes exhibited significantly increased mRNA expression of USP7, TLR4, HMGB1, and the NF-κB subunit p65 (Figure 3b). Following 24 h of co-culture, a duration chosen to reflect subsequent protein accumulation, the protein levels of USP7, TLR4, HMGB1, and p65 in monocytes were also markedly upregulated (Figure 3c and d). ELISA analysis of the co-culture supernatants revealed time-dependent increases in TNF-α, IL-6, IL-1β, and HMGB1 concentrations at both 10 and 24 h (Figure 3e). Notably, immunofluorescence showed that nuclear translocation of p65 in monocytes was not significantly increased at the early 10-hour time point but was clearly enhanced after 24 h of co-culture (Figure 3f), suggesting that NP cells robustly activate the NF-κB signaling pathway and DAMP-associated responses in monocytes.

We next examined the effects of activated monocytes on NP cell function. CCK-8 assays showed that, compared with NP cells in monoculture, the viability of NP cells co-cultured with monocytes began to show a statistically significant reduction at 24 h and continued to decline thereafter (Figure 3g), indicating the cytotoxic potential of monocyte-derived inflammatory factors. Further qPCR analysis showed that after 12 h of co-culture, the mRNA levels of inflammatory markers IL-1β, IL-6, and cyclooxygenase-2 were significantly increased in NP cells, suggesting the onset of an early inflammatory response (Figure 3h). Additionally, oxidative stress was evident, as indicated by reduced expression of the antioxidant enzyme SOD2 and increased intracellular reactive oxygen species (ROS) levels (Figure 3i). Collectively, these data indicate that NP cells undergo a time-dependent inflammatory and oxidative stress response upon exposure to activated monocytes.

### Activation of the USP7–p65 axis in monocytes is directly associated with stress responses in NP cells

**Fig. 4:**
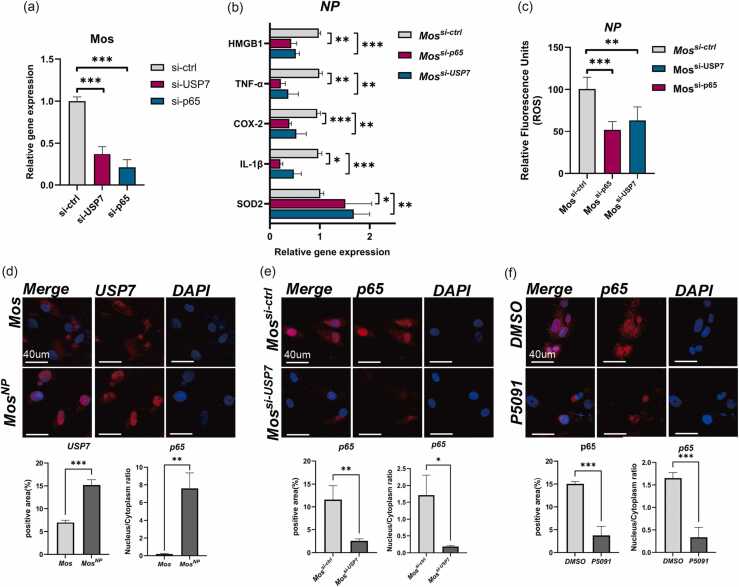
*The USP7–p65 axis in monocytes is directly associated with NP cell stress.* (a) Monocytes were pretreated with a transduction enhancer for 30 min, followed by standard siRNA transfection targeting USP7 or p65. Knockdown efficiency was validated by qPCR (*n* = 3). (b) qPCR analysis of TNF-α, HMGB1, IL-1β, COX-2, and SOD2 mRNA levels in NP cells after 12-hour co-culture with monocytes transfected with control siRNA (si-ctrl), si-USP7, or si-p65 (*n* = 3). (C) Intracellular ROS levels in NP cells were assessed using the DCFH-DA probe following 12-hour co-culture with monocytes transfected with si-ctrl, si-USP7, or si-p65 (*n* = 3). (d) Immunofluorescence analysis of USP7 expression and subcellular localization in monocytes under monoculture or co-culture conditions. Scale bar: 40 µm. Quantification of mean USP7 fluorescence intensity and nuclear-to-cytoplasmic (N/C) ratio is shown below. (e) Immunofluorescence analysis of p65 expression and localization in monocytes transfected with si-ctrl or si-USP7 under co-culture conditions. Scale bar: 40 µm. Quantitative analysis of mean p65 intensity and N/C ratio is provided. (f) Immunofluorescence analysis of p65 expression and localization in monocytes pretreated with DMSO (0.05%) or the USP7 inhibitor P5091 (5 μM) for 3 h prior to co-culture. Scale bar: 40 µm. Quantitative analysis of mean p65 intensity and N/C ratio is shown. Data are presented as mean ± SD. Statistical significance: *P < 0.05; **P < 0.01; ***P < 0.001.

To clarify the role of the USP7–p65 axis in monocytes, we used an optimized transfection reagent (NATE™, InvivoGen) to improve siRNA delivery. Knockdown of USP7 and p65 was confirmed by qPCR (Figure 4a). Monocytes transfected with control, USP7, or p65 siRNA were co-cultured with NP cells for 12 h, and NP cells were analyzed for inflammatory and oxidative stress markers. Co-culture with p65-silenced monocytes significantly reduced NP expression of TNF-α, HMGB1, IL-1β, and COX-2, while SOD2 expression increased (Figure 4b). Similar trends were observed with USP7-silenced monocytes, though the reduction in inflammatory genes was milder and SOD2 elevation was more pronounced (Figure 4b). Both knockdowns also reduced ROS accumulation in NP cells (Figure 4c), indicating that USP7 or p65 inhibition in monocytes mitigates NP cell stress responses.

Immunofluorescence showed that NP stimulation enhanced USP7 expression and nuclear localization in monocytes (Figure 4d). USP7 knockdown suppressed both p65 expression and its nuclear translocation (Figure 4e). Similarly, P5091(5 μM), a small-molecule inhibitor that suppresses the deubiquitinating function of USP7,^14^ significantly reduced p65 protein expression and nuclear localization (Figure 4f). These findings suggest that USP7 regulates p65 activity through deubiquitination in response to NP immunogenicity. Targeting USP7 in monocytes may thus alleviate NP inflammation and oxidative stress, highlighting its potential as a therapeutic target in IDD.

### Monocytes enhance promoter activity of TNF-α, HMGB1, and IL-1β via the USP7–p65 axis in response to NP immunogenicity

**Fig. 5:**
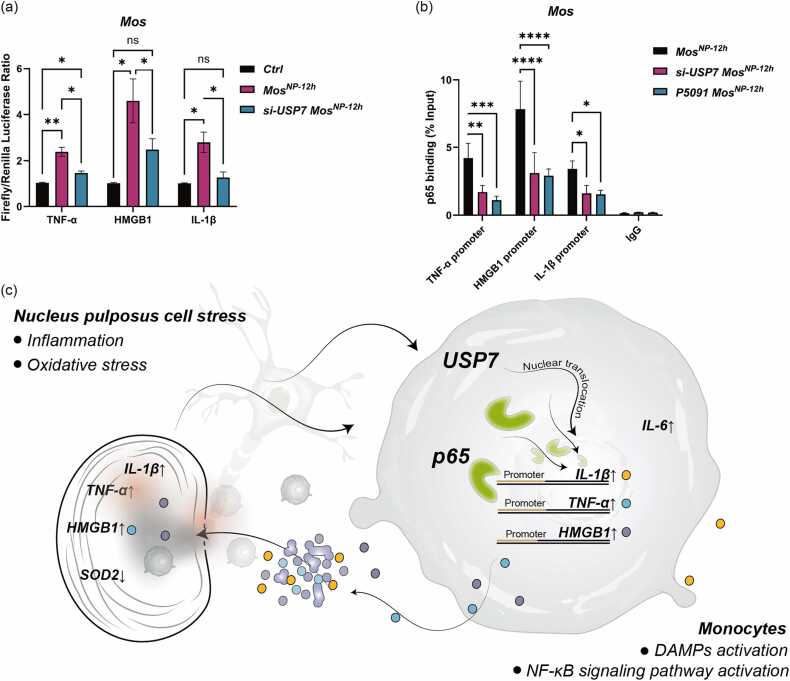
*Monocytes promote TNF, IL-1β, and HMGB1 promoter activity via USP7 in response to NP immunogenicity***.** (a) The ∼1 kb promoter regions of human TNF-α, IL-1β, and HMGB1 were cloned upstream of the firefly luciferase gene in the psiCHECK™−2 dual-luciferase reporter vector, with Renilla luciferase serving as an internal control. Reporter constructs were transfected into THP-1 monocytes for 24 h, followed by three conditions: (i) unstimulated monocytes (Ctrl); (ii) co-culture with NP cells for 12 h (Mos^NP-12h^); and (iii) USP7 knockdown monocytes co-cultured with NP cells for 12 h (siUSP7 Mos^NP-12h^). Luciferase activity was quantified using the Dual-Luciferase® Reporter Assay System (Promega). Firefly signals were normalized to Renilla, and promoter activity was expressed relative to the Ctrl group. (b) ChIP-qPCR was performed to assess p65 enrichment at the promoter regions of TNF-α, HMGB1, and IL-1β in monocytes under NP stimulation. THP-1 cells were pretreated with a transfection enhancer and divided into three experimental groups: (1) co-cultured with NP cells for 12 h (Mos^NP-12h^); (2) transfected with USP7 siRNA prior to co-culture (si-USP7 Mos^NP-12h^); and (3) pretreated with the USP7 inhibitor P5091 (5 μM, 3 h) before co-culture (P5091 Mos^NP-12h^). Chromatin was immunoprecipitated using an anti-p65 antibody, and promoter-specific DNA enrichment was quantified by qPCR. Results were expressed as a percentage of input (% input). (c) Schematic illustration of the proposed mechanism by which USP7 mediates monocyte activation in response to the immunogenicity of NP cells. Upon stimulation by NP-derived immunogenic factors, monocytes exhibit upregulation and nuclear translocation of USP7, which enhances the transcriptional activity of p65 through a deubiquitination-dependent mechanism. This process leads to activation of the NF-κB signaling pathway, resulting in increased expression of TNF, IL-1β, and HMGB1 in monocytes. These pro-inflammatory mediators subsequently induce inflammatory and oxidative stress responses in NP cells, as evidenced by elevated expression of TNF, IL-1β, and HMGB1, along with decreased expression of the antioxidant enzyme SOD2. This establishes a feed-forward loop of inflammation and oxidative stress between monocytes and NP cells. Data are presented as mean ± standard deviation (SD), *n* = 3. Statistical significance was assessed by two-way ANOVA: *P < 0.05, **P < 0.01, ***P < 0.001.

We next investigated the role of USP7 in regulating the transcriptional activation of inflammatory genes using dual-luciferase reporter assays. Monocytes were transfected with luciferase reporter plasmids containing the promoter regions of TNF-α, HMGB1, or IL-1β, and stimulated with NP cell–conditioned medium to mimic an immune-activating environment. Under monoculture conditions, promoter activities remained at baseline. However, following 12 h of stimulation, promoter activities were significantly elevated: TNF-α by ∼2.05-fold, HMGB1 by ∼4.26-fold, and IL-1β by ∼2.3-fold relative to controls (Figure 5a). These findings suggest that USP7 contributes to the upregulation of inflammatory gene transcription in monocytes in response to NP-derived immunogenic cues.

To determine whether this USP7-mediated promoter activation is dependent on p65, we performed ChIP-qPCR using an anti-p65 antibody to assess p65 binding to the promoter regions of TNF-α, HMGB1, and IL-1β. Compared to the NP co-culture group, USP7 knockdown markedly reduced p65 enrichment at all three promoters (Figure 5b). Notably, monocytes pretreated with P509 also showed a similar reduction in p65 binding to these promoter regions. These results indicate that USP7 promotes p65-mediated transcriptional activation by preventing its degradation, thereby enhancing the expression of DAMP-related genes in monocytes upon NP stimulation. This USP7–p65 axis may thus amplify the inflammatory and oxidative stress response in NP cells (Figure 5c).

## Discussion

This study reveals a mechanism by which the immunogenicity of the NP, following the loss of its immune privilege, drives crosstalk between monocytes and NP cells. A pivotal event in this process is the upregulation of USP7 in monocytes. Immunogenic exposure to NP tissue activates peripheral monocytes, inducing their rapid activation and infiltration, which in turn exacerbates inflammation and oxidative stress in NP cells, thereby establishing a vicious cycle. Monocytes exposed to NP components display activation of DAMP-related signaling and the USP7–p65 axis, characterized by marked upregulation of USP7 expression accompanied by pronounced nuclear translocation. Through its deubiquitinating activity, USP7 further enhances p65 stability and nuclear localization, thereby promoting transcriptional activation and paracrine release of TNF-α, HMGB1, and IL-1β. These cytokines amplify inflammatory and oxidative stress responses in NP cells, as evidenced by increased expression of TNF-α, HMGB1, IL-1β, and IL-6, downregulation of the antioxidant enzyme SOD2, and accumulation of ROS.

USP7 is a deubiquitinase previously reported to regulate inflammatory signaling pathways within tumor-associated microenvironments.^15^ In tumor cells, USP7 specifically removes ubiquitin moieties from the NF-κB subunit p65, thereby stabilizing p65 and enhancing its transcriptional activity toward pro-inflammatory genes.^16^ Conversely, inhibition of USP7 dampens inflammatory responses: silencing USP7 in endothelial cells suppresses their inflammatory activation and reduces adhesion to monocytes,^17^ while pharmacological inhibition of USP7 activity impairs NLRP3 inflammasome assembly and decreases the production of pro-inflammatory cytokines.^18^ Collectively, these findings indicate that USP7 functions as a positive regulator of inflammatory signaling.

In the present study, NP stimulation rapidly induced USP7 upregulation and nuclear translocation in monocytes, a change likely to enhance activation of NF-κB and other inflammatory pathways, thereby amplifying inflammatory and oxidative stress responses. Dual-luciferase reporter assays and ChIP-qPCR further demonstrated that USP7 in monocytes regulates the downstream targets of p65: USP7 overexpression promoted p65 binding and enrichment at the promoter regions of TNF-α, HMGB1, and IL-1β, markedly increasing their promoter activity and transcriptional levels. Conversely, USP7 inhibition reduced p65 binding and attenuated its transcriptional activation. These results indicate that USP7 amplifies pro-inflammatory cytokine production by stabilizing and enhancing p65 activity. This mechanism provides new insight into NP immunogenicity-driven inflammation, positioning USP7 as an “amplifier” of inflammatory signaling that drives excessive release of pro-inflammatory mediators from monocytes, thereby exacerbating IDD.

As early as 2007, Geiss et al implanted autologous, non-degenerated porcine NP subcutaneously into pigs for 7 days and observed a significant increase in activated B and T lymphocytes.^8^ Subsequently, Murai et al confirmed NP immunogenicity from another perspective: transplantation of non-degenerated rat NP tissue into wild-type and immunodeficient mice resulted in significantly higher NP cell survival in immunodeficient mice, implicating macrophages as key mediators.^19^ However, the molecular sources of this immunogenicity remain poorly understood. Krock et al demonstrated that stimulation of NP cells with a 30 kDa fibronectin fragment significantly increased pro-inflammatory cytokine secretion,^20^ suggesting fibronectin fragments as one potential source of immunogenicity. Similarly, Shah et al reported that HMGB1 released from degenerated NP cells acts as an effective DAMP, promoting NP cell inflammation and tissue degradation.^21^ Nevertheless, additional evidence is required to fully elucidate NP immunogenicity, and further studies are warranted to explore the upstream signals within NP tissue that activate monocytes.

This study has several limitations. First, we primarily examined the acute responses of monocytes to NP stimulation, without fully evaluating the potential roles of other immune cell types, such as T lymphocytes, in disc-related immune responses. Although we employed an in vitro co-culture system to simulate the in vivo environment, this model cannot fully recapitulate the complex interactions between cells and extracellular matrix components within the native disc microenvironment. Second, the upstream signals within NP tissue that activate monocytes remain incompletely defined. Further research is needed to delineate these mechanisms.

## Methods

### Experimental animals and NP tissue collection

Three-month-old male Sprague-Dawley rats (250-300 g) were used. All animal experiments were performed in accordance with the guidelines of the Animal Experiment Ethics Committee and were approved by the Ethics Committee of Beijing Longan Laboratory Animal Center (Approval No. BJLongan-L-L-003). To establish the disc degeneration model, under general anesthesia (2% pentobarbital sodium, 30 mg/kg, intraperitoneal), and after local disinfection, a 21 G needle was vertically inserted into the center of the Co6/7, Co7/8, and Co8/9 caudal intervertebral discs to puncture the NP, left in place for 30 s, and then slowly withdrawn (the control group discs were only punctured superficially in the skin).

Postoperatively, rats received routine care and antibiotics to prevent infection. After 4 weeks, MRI evaluation (Siemens Healthineers, MAGNETOM Skyra 3 T) confirmed disc degeneration, and the degenerated discs were harvested. The tail skin and subcutaneous tissue were opened along the sagittal plane, the superior cartilaginous endplate was lifted, and a curette was used to carefully isolate the NP tissue. Part of the NP tissue was fixed in 4% paraformaldehyde for histological analysis, and another part was immediately minced on ice and collected for subsequent molecular experiments.

### Monocyte–NP cell co-culture system and cell transfection

THP-1 monocytes were obtained from the Cell Resource Center of the Institute of Basic Medical Sciences, Chinese Academy of Medical Sciences. Human immortalized NP cells (CP-H097Y) were purchased from Wuhan Pricella Biotechnology Co., Ltd. (Wuhan, China). A co-culture system was established using Transwell inserts (0.4 µm pore polycarbonate membrane): NP cells were placed in the lower chamber and monocytes in the upper chamber. If transfection was required, monocytes were transfected in monoculture first, then the co-culture system was set up.

Monocytes were treated with a transfection enhancer (NATE™-Transfection & Transduction Enhancer, InvivoGen) for 30 min prior to transfection to improve transfection efficiency.^22^ Using Lipofectamine 3000 (Invitrogen, L3000150), USP7-targeting siRNA (si-USP7) or a non-specific negative control siRNA (si-ctrl) was transfected into monocytes according to the manufacturer’s protocol. Six hours after transfection, the medium was replaced with fresh medium. USP7 knockdown efficiency was verified by qPCR.

### Single-cell RNA sequencing data acquisition and analysis

We analyzed publicly available single-cell RNA sequencing datasets obtained from the GEO database.^23^ Data processing was performed using the Seurat package (v4.0.6) in R. Following data download, we conducted standard preprocessing steps, including quality control, normalization, and dimensionality reduction of the raw expression matrices. Cell clusters were classified and annotated based on the expression patterns of established marker genes for major intervertebral disc cell types (e.g., ACAN and COL2A1 for NP cells) and immune cells (e.g., CD86, CD68, and CD163 for monocytes/macrophages), combined with reference to published atlases of human intervertebral disc single-cell profiles. Monocyte/macrophage clusters were subsequently identified and extracted for downstream analysis when analyzing immune cell responses, whereas NP cell clusters were selected when focusing on NP cell–specific responses.

Differentially expressed genes (DEGs) were identified using Seurat’s FindMarkers function with the Wilcoxon rank-sum test, applying the following thresholds: adjusted *P* < 0.05 and |log₂ fold change| > 1. To investigate the biological significance of these DEGs, we performed Gene Ontology and Kyoto Encyclopedia of Genes and Genomes (KEGG) pathway enrichment analyses using the clusterProfiler package (v4.2.2). These analyses allowed us to explore relevant biological processes, cellular components, molecular functions, and signaling pathways associated with monocyte/macrophage activity in degenerated disc tissue.

### Western blot analysis

Total protein was extracted from cells using RIPA lysis buffer (with protease inhibitors, Thermo Scientific, 78440). Protein concentration was measured by the BCA method (Thermo Scientific, 23225). Equal amounts of protein were separated by SDS-PAGE and transferred onto PVDF membranes. Membranes were blocked with a rapid blocking buffer for 5 min (Bio-Rad, 12010020), then incubated with primary antibodies (CD11b, TLR4, USP7, CD86, HMGB1, GAPDH) overnight at 4 °C. The next day, membranes were incubated with HRP-conjugated secondary antibodies for 1 h at room temperature, then developed using ECL substrate (Thermo Scientific, 32209) and imaged. Band intensities were quantified using ImageJ and normalized to GAPDH.

The following antibodies were used in this study: CD11b polyclonal antibody (Abcam, Cat# ab133357); TLR4 polyclonal antibody (Thermo Scientific Cat# PA5-23124); USP7 monoclonal antibody (Proteintech, Cat# 66514-1-Ig); CD86 polyclonal antibody (Proteintech, Cat# 30691-1-AP); HMGB1 polyclonal antibody (Proteintech, Cat# 10829-1-AP); and GAPDH monoclonal antibody (Proteintech, Cat# 60004-1-Ig).

### Quantitative real-time PCR (qPCR)

Total RNA was extracted using the RNeasy Plus Mini Kit (Qiagen) and its concentration and purity were measured with NanoDrop. cDNA was synthesized using the PrimeScript™ RT reagent kit (Takara). Quantitative PCR was performed using iTaq™ Universal SYBR® Green Supermix (Bio-Rad) on a CFX96 real-time PCR system. The cycling conditions were: 95 °C for 30 s, followed by 40 cycles of 95 °C for 5 s and 60 °C for 30 s. Gene expression was normalized to GAPDH and relative expression levels were calculated using the 2^−ΔΔCt^ method. Each sample was run in triplicate. All primer sequences are provided in Table 2.

**Table 2:** Primer sequences used for PCR amplification.

Species	Genes	Primer Sequence	Size (bp)
Rat	CD11b	Forward: GCTCCTCAAGGTCGTTGTGA Reverse: AGATGGCGTACTTCACAGGC	92
Human	USP7	Forward: CCCTCCGTGTTTTGTGCGA Reverse: AGACCATGACGTGGAATCAGA	133
Human	TLR4	Forward: AGACCTGTCCCTGAACCCTAT Reverse: CGATGGACTTCTAAACCAGCCA	147
Human	HMGB1	Forward: TGCTGCTCCCTCTTTGGAG Reverse: CCCATGTTTAGTTATTTTTCGTGC	84
Human	SOD2	Forward: GGAAGCCATCAAACGTGACTT Reverse: CCCGTTCCTTATTGAAACCAAGC	116
Human	COX-2	Forward: CTGGCGCTCAGCCATACAG Reverse: CGCACTTATACTGGTCAAATCCC	94
Human	IL-1β	Forward: ATGATGGCTTATTACAGTGGCAA Reverse: GTCGGAGATTCGTAGCTGGA	132
Human	IL-6	Forward: ACTCACCTCTTCAGAACGAATTG Reverse: CCATCTTTGGAAGGTTCAGGTTG	149
Human	P65	Forward: CCCACGAGCTTGTAGGAAAGG Reverse: GGATTCCCAGGTTCTGGAAAC	96
Human	TNF-α promoter	Forward: GCTCATGGGTTTCTCCACCA Reverse: GTGTGCCAACAACTGCCTTT	101
Human	HMGB1 promoter	Forward: ACTCGCGTCTCCACTAGGAA Reverse: GCTCACGTCTTCACCTTCCC	120
Human	IL-1βpromoter	Forward: GGTGTTCCTACACTCCAGGC Reverse: AACCGAGACACCAGCAAAGT	111

### Immunofluorescence staining

Rat tissue samples were fixed in 4% paraformaldehyde, embedded in paraffin, and sectioned at a thickness of 4 µm. Sections were deparaffinized in xylene, rehydrated through a graded ethanol series, and subjected to antigen retrieval using 10 mM sodium citrate buffer (pH 6.0). Following retrieval, sections were permeabilized with 0.3% Triton X-100 for 10 min and then blocked with 5% bovine serum albumin for 1 h at room temperature.

Tissue sections were incubated with CD11b primary antibodies (Abcam, Cat# ab133357) overnight at 4 °C, followed by incubation with fluorescently labeled secondary antibodies for 1 h at room temperature the next day. Nuclei were counterstained with DAPI, and the sections were mounted with coverslips. Fluorescence images were captured using a fluorescence microscope.

Monocytes were seeded onto poly-L-lysine-coated slides and allowed to adhere at room temperature for 45 min. Cells were then fixed with 4% paraformaldehyde for 10 min and permeabilized with 0.2% Triton X-100 for 5 min. To block non-specific binding, cells were incubated with 5% bovine serum albumin for 30 min at room temperature. Subsequently, cells were incubated overnight at 4 °C with USP7 primary antibody (Proteintech, Cat# 66514-1-Ig). The following day, cells were incubated with fluorescently labeled secondary antibodies for 1 h at room temperature, followed by nuclear counterstaining with DAPI. After thorough PBS washes, slides were mounted and examined under a fluorescence microscope for imaging and analysis.

### Dual-luciferase reporter gene assay

To evaluate the transcriptional activity of the TNF-α and HMGB1 promoters, dual-luciferase reporter vectors were constructed. Approximately 1000 bp of the upstream genomic sequences of the human TNF-α and HMGB1 promoter regions were amplified and cloned into the multiple cloning site upstream of the Renilla luciferase gene in the psiCHECK™−2 vector (Promega, USA) to drive its expression; Firefly luciferase served as an internal control reporter gene under an independent promoter in the same vector. After successful construction, the plasmids were transfected into monocytes. Twenty-four hours post-transfection, monocytes were stimulated with NP-conditioned medium for 12 h. Luciferase activity was then measured using the Dual-Luciferase® Reporter Assay System (Promega), and Renilla and Firefly signals were read. Promoter activity was calculated as the Renilla/Firefly ratio and normalized to the unstimulated control group. All experiments were repeated 3 times.

### Enzyme-Linked Immunosorbent Assay (ELISA)

Cell culture supernatants were collected, and ELISA kits were used to measure the concentrations of TNF-α and HMGB1, following the manufacturer’s instructions.

### ChIP-qPCR assay

To evaluate the binding of NF-κB p65 to the promoter regions of inflammation-related genes, chromatin immunoprecipitation (ChIP) followed by quantitative PCR (ChIP-qPCR) was performed in monocytes stimulated by NP cells. Briefly, THP-1 monocytes were either cultured alone or co-cultured with NP cells for 12 h. ChIP was conducted using the SimpleChIP® Enzymatic Chromatin IP Kit (Cell Signaling Technology, Cat# 9003S), following the manufacturer’s protocol. Cells were cross-linked with 1% formaldehyde at room temperature for 10 min and quenched with 125 mM glycine. Chromatin was then digested with micrococcal nuclease and sheared by sonication to obtain DNA fragments ranging from 150 to 900 bp. Immunoprecipitation was performed using 5 μg of anti-p65 antibody (Cell Signaling Technology, Cat# 8242) or normal rabbit IgG as a negative control. After elution and reverse crosslinking, DNA was purified and subjected to real-time quantitative PCR using promoter-specific primers targeting TNF-α, HMGB1, and IL-1β. The results were normalized to input DNA and expressed as a percentage of input (% input). Primer sequences are listed in Table 2.

### Statistical analysis

All experiments were independently repeated at least three times. Data are expressed as mean ± standard deviation (Mean ± SD). Statistical analysis was performed using GraphPad Prism 8.0. Comparisons between two groups were made using independent sample t-tests, and comparisons among multiple groups were made using one-way analysis of variance (ANOVA) with Tukey’s post hoc test for pairwise comparisons. A *P* value < 0.05 was considered statistically significant.

## Funding and support

This research was funded by the 10.13039/501100001809National Natural Science Foundation of China (82474542) and the High-Level Traditional Chinese Medicine Hospital Construction Project of Wangjing Hospital, 10.13039/501100005892China Academy of Chinese Medical Sciences (WJZJ-202318).

## Author contributions

CYG and TZ designed the study and revised the manuscript. PF and XLC carried out the experiments. PF, JHG, and YC assembled and analyzed the data. The article was written by PF. All authors approved the final version of the manuscript.

## CRediT authorship contribution statement

**Peng Feng:** Writing – original draft, Investigation, Formal analysis. **Xuelei Chu:** Investigation. **Ying Che:** Formal analysis. **Jinghua Gao:** Formal analysis. **Chunyu Gao:** Writing – review & editing, Supervision, Methodology, Conceptualization. **Ting Zhang:** Writing – review & editing, Supervision, Conceptualization.

## Declaration of Interest

The authors declare that they have no known competing financial interests or personal relationships that could have appeared to influence the work reported in this paper.

## Data Availability

Data will be made available on request.
