# Supplementary material for: Monocyte USP7-p65 axis mediates immune responses to the immunogenicity of nucleus pulposus
Source: Cell Stress Chaperones. 2025 Sep 12;30(6):100114. doi: 10.1016/j.cstres.2025.100114 (PMC12495435; doi:10.1016/j.cstres.2025.100114)
Supplement: Supplementary file 1 — Supplementary material [file mmc1.docx]

The original Western blot images corresponding to the figures in the main text are provided below. Red boxes indicate the regions used in the main figures.


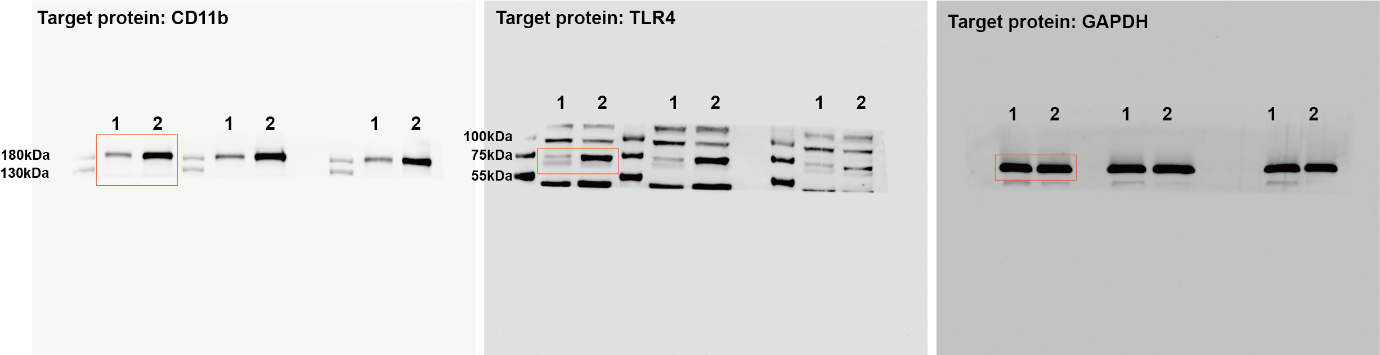


**Supplementary Figure 1**. Raw Western blot images. The original blot for Figure 1D (CD11b, TLR4, GAPDH). Regions used in the main figures are indicated by red boxes.


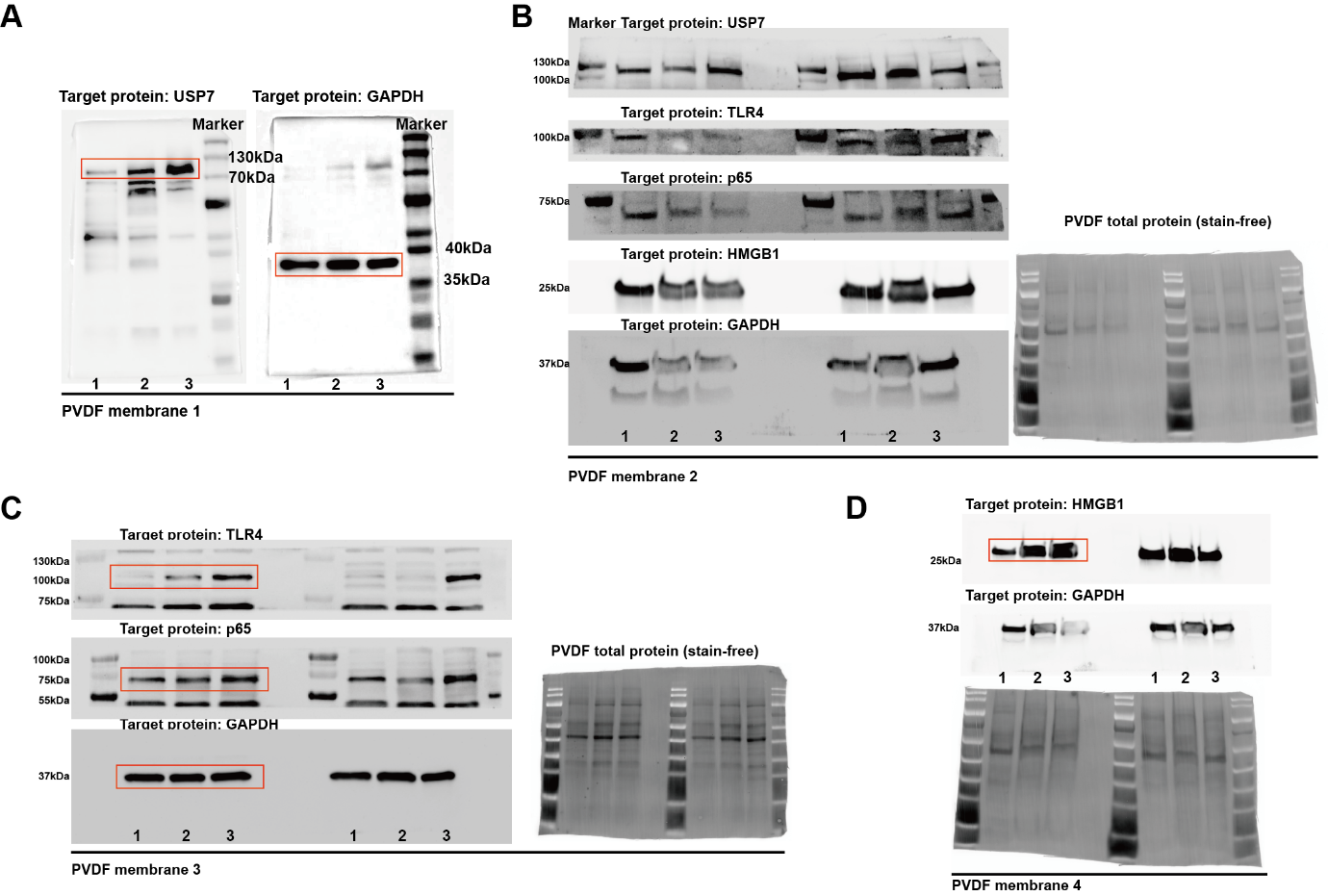


**Supplementary Figure 2**. Raw Western blot images.

(A) The original blot for Figure 3C (USP7, GAPDH) in PVDF membrane 1.

(B) The original blot for Figure 3C (USP7, TLR4, p65, HMGB1, GAPDH) in PVDF membrane 2.

(C) The original blot for Figure 3C (USP7, TLR4, p65, GAPDH).

(D) The original blot for Figure 3C (HMGB1, GAPDH).

Regions used in the main figures are indicated by red boxes.
